# Relationship between Plasma Lipoprotein-Associated Phospholipase A2 Concentrations and Apolipoprotein in Stable Coronary Artery Disease Patients

**DOI:** 10.1155/2020/8818358

**Published:** 2020-09-24

**Authors:** Yang Ling, Shengxing Tang, Yuhan Cao, Cong Fu

**Affiliations:** ^1^Department of Cardiology, Yi Ji Shan Hospital Affiliated to Wan Nan Medical College, China; ^2^Department of Nephrology, Yi Ji Shan Hospital Affiliated to Wan Nan Medical College, China; ^3^Key Laboratory of Non-Coding RNA Transformation Research of Anhui Higher Education Institution (Wan Nan Medical College), China

## Abstract

**Background:**

Increasing evidence states that the plasma lipoprotein-associated phospholipase A2 (Lp-PLA2) levels and apolipoprotein particles are regarded as the risk maker for cardiovascular heart disease. Nevertheless, the issue about whether Lp-PLA2 is associated with apolipoprotein particles in individuals who have been diagnosed as stable coronary artery disease (CAD) remains largely unexplored.

**Method:**

All 569 participants engaged in this research, who never took lipid-lowering drugs, had been divided into groups by the coronary angiography (CAG), namely, stable CAD: *n* = 291; non-CAD: *n* = 278. The results concerning Lp-PLA2 levels were calculated by Elisa Kit, while apolipoprotein particles were measured by the department of laboratory.

**Results:**

The plasma concentration of Lp-PLA2 was remarkably higher in stable CAD group than the non-CAD group (136.0 ± 60.5 ng/mL vs. 113.2 ± 65.6 ng/mL, *P* < 0.001). Pearson correlation analyses explained the plasma Lp-PLA2 concentration was correlated with apoB (*r* = 0.390, *P* < 0.001) and apoB/apoA1 (*r* = 0.450, *P* < 0.001), not associated with apoA1 (*r* = −0.099, *P* = 0.101). Conversely, the association remains unobserved among non-CAD patients except apoA1. Moreover, multiple linear regression revealed the relations between Lp-PLA2 concentrations and apoB (*β* = 0.390, *P* < 0.001), as well as apoB/apoA1 (*β* = 0.450, *P* < 0.001), but not apoA1 (*β* = −0.099, *P* = 0.121). After adjustment for several risk factors regarding CAD, like hypertension, gender, smoking, age, and diabetes mellitus, there had still been positive associations between the Lp-PLA2 concentration and apoB (*β* = 0.364, *P* < 0.001), as well as apoB/apoA1 (*β* = 0.390, *P* < 0.001).

**Conclusion:**

The plasma levels of Lp-PLA2 provide positively a key link with apoB, apoB/apoA-1 among stable CAD, denoting the communication between Lp-PLA2 and apolipoprotein particles in the state of CAD.

## 1. Introduction

A growing number of people are currently suffering from a range of major clinical heart and circulatory disease conditions, including coronary artery disease (CAD), heart failure, peripheral arterial disease, and cerebrovascular disease [1]. CAD is the leading cause of mortality and morbidity, which leads to immense health and economic burdens in the United States even globally [1]. Although the pathogeneses of CAD are interconnected, growing evidence points to a vital role of inflammation in the disease progression [2–4]. Lipoprotein-associated phospholipase A2 (Lp-PLA2), which is characterized by a Ca+ independent enzyme, originally tends to be defined as platelet-activating factor acetylhydrolase (PAF-AH) and is principally secreted by inflammatory cells (macrophages) [5]. Lp-PLA2 is a member of the PLA2 superfamily, characterized by the specific ability to hydrolyze the sn-2 position of phospholipids [6, 7]. It means that Lp-PLA2 could catalyze the hydrolysis of sn-2 residue, resulting in producing lysophosphatidylcholine (lyso-PC) and nonesterified fatty acids that are associated with oxidative stress and inflammation response [8]. Due to the proinflammatory effects and various studies, which had illustrated that the Lp-PLA2 was correlated with a wide range of cardiovascular diseases [9–11], Lp-PLA2 is gradually identified as a reliable biomarker for the risk of clinical cardiovascular events.

Generally, the plasma circulating Lp-PLA2 binds to lipoproteins, whereby approximately 70% to 80% and 20% to 30% of the total activity have been carried by low-density lipoprotein (LDL) and high-density lipoprotein (HDL), respectively [6]. Furthermore, because each LDL particle contains one molecule of apolipoprotein B-100, the majority of apolipoprotein B (apo-B) would indirectly test the number of LDL particles. Apolipoprotein A-1 (apoA-1) comprises nearly 70% of the apolipoproteins on the HDL particles, allowing an effective calculation of HDL concentration. Meanwhile, there is a definite connection between Lp-PLA2 level and LDL-cholesterol concentration whereas negatively related to HDL-cholesterol concentration [12, 13]. Consequently, understanding the relation between Lp-PLA2 and apolipoprotein may suggest several potential determinants with regard to the risk of CAD. In fact, the previously published research had revealed that the ratio of apoB/apoA-1 appeared to be more significantly associated with the extent of coronary artery lesions in Chinese diabetics [14]. However, to some extent, the association between Lp-PLA2 and apolipoprotein remains disclosed in stable coronary artery disease patients. Accordingly, the purpose of the present study is to investigate the potential association between the Lp-PLA2 activity and apolipoprotein among stable CAD group, where all participants never received lipid-lowering drugs orally before.

## 2. Methods

### 2.1. Study Design and Population

This study was approved by the Ethics Committee of Yi Ji Shan Hospital and complied with the Declaration of Helsinki. Informed written consent was obtained from all participants enrolled in this study. 291 stable coronary artery disease patients confirmed by coronary angiography and 278 non-CAD patients in the Department of Cardiology, Yi Ji Shan Hospital from Jul. 2017 to Dec. 2018 were recruited in this study. Individuals with myocardial infarction, unstable angina, heart failure (left ventricular ejection fraction<45%), severe kidney, and/or liver dysfunction, cancer, infectious disease, inflammatory disease, autoimmune diseases, treatment history of lipid-lowering drugs were excluded from the research. The lifestyle risk factors of these participants were determined by several questionnaires. The CAD was defined as the presence of at least one lesion with more than 50% luminal stenosis in the coronary arteries, with a diameter of greater than 2 mm. Non-CAD was defined as patent arteries or insignificantly diseased coronary arteries (less than 50% luminal stenosis) examined through coronary angiography (CAG). The definition of myocardial infarction was described previously [15].

### 2.2. Laboratory Testing and Measurement of Plasma LP-PLA2 Concentration

Blood samples were obtained in all patients from the peripheral vein 12-24 hours after CAG. EDTA-containing tube was used to collect the blood sample. Plasma was collected after 500 g centrifuged for 20 minutes in 4°C. Plasma was stored in -80°C. Lp-PLA2 Elisa Kit (X-Y Biotechnology) was used to determine the plasma Lp-PLA2 concentration according to the manufacturer's protocol. The measurement of biochemical message was performed in the Department of Laboratory, Yi Ji Shan Hospital 24 hours after hospitalized.

### 2.3. Statistical Analysis

Continuous variables were expressed as mean ± standard deviation. Categorical variables were expressed as number (%). To compare the clinical biochemical message and Lp-PLA2 levels between stable CAD and non-CAD groups, Student's *t*-test was used. Categorical variables were compared by *χ*^2^. The relationships between the parameters were assessed by Pearson correlation. The associations between the Lp-PLA2 and related variables were evaluated by multivariable linear regression analysis. The value of *P* < 0.05 was regarded as statistical significance. Statistical processing was conducted through SPSS statistical software (Version 17.0).

## 3. Results

### 3.1. Baseline Characteristics

**Figure 1:**
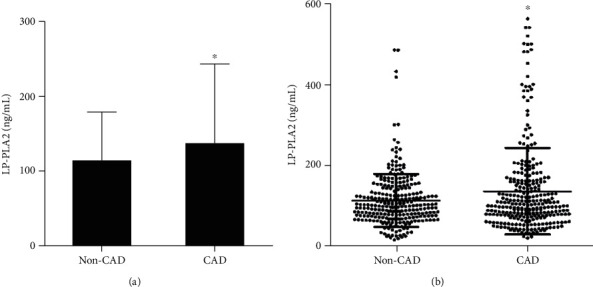
(a) The plasma concentration of LP-PLA2 in stable CAD and non-CAD was shown by histogram. (b) The data of LP-PLA2 between stable CAD and non-CAD patients were depicted by the scatter diagram. Those figures displayed that the plasma concentration of LP-PLA2 was remarkedly higher in stable CAD group than the non-CAD group (^∗^*P* < 0.001 vs. non-CAD).

The baseline clinical features and serum biochemical parameters of the participants were displayed in Table 1. Individuals with stable CAD tended to be intensely older than non-CAD patients (*P* < 0.001), and the incidence of hypertension or diabetes was extremely higher than the non-CAD group (*P* < 0.05). Additionally, the plasma concentration of Lp-PLA2 was remarkably higher in stable CAD group than the non-CAD group (136.0 ± 60.5 ng/mL vs. 113.2 ± 65.6 ng/mL, *P* < 0.001, Figures 1(a) and 1(b)). None of the other data had been statistically significant between the two groups.

### 3.2. The Relations between Plasma LP-PLA2 Concentration and Apolipoprotein

**Figure 2:**
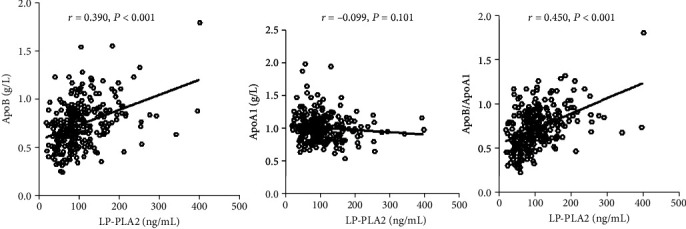
The relationship between plasma concentration of LP-PLA2 and lipoprotein in stable CAD patients. The correlation diagram showed that the plasma LP-PLA2 concentration was linked with ApoB and ratio of ApoB/ApoA1 in stable CAD patients.

**Figure 3:**
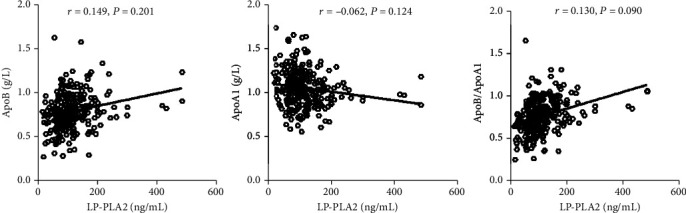
The relationship between plasma concentration of LP-PLA2 and lipoprotein in non-CAD patients. The correlation diagram showed that the plasma concentration of LP-PLA2 has no significantly related with ApoB, ApoA1, and ratio of ApoB/ApoA1 in non-CAD patients.

Pearson correlation analyses were performed to assess the relationship between Lp-PLA2 and apoA1, apoB, and apoB/apoA1. As revealed in Figure 2, among stable CAD patients, the plasma concentration of Lp-PLA2 was not correlated with apoA1 (*r* = −0.099, *P* = 0.101). However, the plasma Lp-PLA2 concentration was linked with apoB (*r* = 0.390, *P* < 0.001) and apoB/apoA1 (*r* = 0.450, *P* < 0.001). During non-CAD group, as depicted in Figure 3, the plasma Lp-PLA2 concentration was not associated with apoA1 (*r* = −0.062, *P* = 0.124), apoB (*r* = 0.149, *P* = 0.201), and apoB/apoA1 (*r* = 0.130, *P* = 0.090).

As shown in Table 2, multiple linear regression further revealed the relations between Lp-PLA2 concentrations and apoB (*β* = 0.390, *P* < 0.001) as well as apoB/apoA1 (*β* = 0.450, *P* < 0.001), but not apoA1 (*β* = −0.099, *P* = 0.121). After adjustment for several risk factors regarding CAD, like hypertension, gender, smoking, age, and diabetes mellitus, there had still been positive associations between the Lp-PLA2 concentration and apoB (*β* = 0.364, *P* < 0.001) as well as apoB/apoA1 (*β* = 0.390, *P* < 0.001). ApoA1 (*β* = −0.128, *P* = 0.245) showed no relation with plasma LP-PLA2 concentration.

## 4. Discussion

The present study had recruited 569 patients who never took lipid-lowering drugs previously and showed that the plasma concentration of Lp-PLA2 was definitely associated with apoB or apoB/apoA1 in stable CAD subjects. In contrast, no similar consequences were detected among the non-CAD group.

A large number of evidences suggest that inflammation accounts reasonably for the initiation and progression of atherosclerosis and active inflammatory responses might cause ruptured plaque and worsen the risk of coronary thrombosis resulting in various vascular events. The major inflammatory cells comprised of the atherosclerotic lesions are macrophages together with T cells [16, 17]. As mentioned before, the circulating plasma Lp-PLA2, principally secreted by inflammatory cells (macrophages), tends to combine with LDL and HDL, specifically the apoB-containing LDL, as well as apoA-1-containing HDL [18]. Lp-PLA2 could exert the specific proatherogenic role by lyso-PC and modified free fatty acids, the proinflammatory products of Lp-PLA2 activity, which might prompt atherosclerotic plaque progression. It is reported that the thin-cap fibroatheromas and activated plaques abound with the Lp-PLA2 as well as lyso-PC. In addition, an unfavourable feed-forward effect can be related to generate several proinflammatory factors through Lp-PLA2, while enrollment of macrophages, T cells among unstable plaques might further trigger the production of Lp-PLA2 [8]. Of note, it has been proposed that the circulating Lp-PLA2 is remarkably and particularly related to recurrent coronary events in postinfarction patients from the previously published study, which also views the Lp-PLA2 as a promising marker of atherosclerosis events [19]. Some large prospective studies demonstrated the constant relationship between Lp-PLA2 and the risk of coronary heart disease [20]. Moreover, a meta-analysis of lipid-related markers and cardiovascular disease prediction revealed that the addition of information on the connection of apoB and apoA-1, or Lp-PLA2 concentrations to risk scores comprising total cholesterol and HDL-c, leads to mild improvement in predicting the cardiovascular disease [21]. Another research had identified that the higher plasma Lp-PLA2 concentrations are linked to the greater incidence of CAD, independently of traditional CAD risk factors, indicating Lp-PLA2 may impose a significant action in the development of atherosclerosis, and be a novel biomarker of dreadful vascular events at follow-up [22]. White et al. found that a larger decrease in Lp-PLA2 activity among the Long-term Intervention with Pravastatin in Ischemic Disease (LIPID) is related to fewer cardiovascular events, independent of change in LDL cholesterol, although higher Lp-PLA2 activity is associated with greater risks of adverse vascular events, denoting that the Lp-PLA2 may be a potential therapeutic goal for CAD [23]. Additionally, Khuseyinova et al. stated that increased Lp-PLA2 levels are correlated with stable CAD, independently of diverse biomarkers, which indicated that the Lp-PLA2 provides more insights into the risk of CAD [24]. In the current paper, we had uncovered the definite correlations between plasma Lp-PLA2 concentration and apoB, apoB/apoA-1, which was in accord with previous research.

It is well established that the retained apoB-containing lipoproteins is the initial step of atherogenesis. The process of subendothelial retention could be modulated and contributes to serial inflammatory responses. Typically, monocyte-derived macrophages would develop into foam cells via swallowing the apoB lipoproteins. Meanwhile, the inflammatory responses caused by macrophages, T cells, and relevant inflammatory cells in turn promote the atherogenesis [25]. Accordingly, the process about subendothelial retention emphasizes the conclusion that inflammation is a result of apoB-containing lipoproteins retention [17]. Although the pathogenesis of CAD is contributed to a wide range of risk factors, lipid-modification has been increasingly viewed as the crucial target, especially decreasing LDL cholesterol based on considerable basic science research, epidemiological studies, and RCTs [26, 27]. It is apparent that apoB is superior to total cholesterol or LDL-c levels with regard to the prognostic value, because apoB can be not only observed on LDL but also bound to the VLDL, as well asl lipoprotein (a) particles [28]. Moreover, the superiority of apoB/apoA-1 has been proposed about estimating the risk of cardiovascular events compared to the traditional lipoprotein ratios [29]. The conclusion from the described meta-analysis reveals that non-HDL-c is inferior to apoB and LDL-c is inferior to non-HDL-c in terms of prediction of vascular risk. Furthermore, the incidence of cardiovascular events can be dramatically attenuated via targeting non-HDL-c with an added 300,000 patients after ten years, while choosing apoB could markedly decrement the unfavorable events by another 500,000 individuals [30]. Rasouli M et al. concluded that apoB/apoA-1, apoB, and Lp (a), as significant risk factors among stable CAD, show a better value compared to other cholesterol ratios, even identifying the apoB/apoA-1 as the best biomarker of CAD in clinical practice [31]. Unfortunately, the relationship between Lp-PLA2 and apolipoprotein particles in the specific state of CAD remains uninvestigated to date. Currently, our data reflected that the plasma concentration of Lp-PLA2 was related to apoB and apoB/apoA1 among stable CAD group, whereas no similar results were conducted during the non-CAD group. Furthermore, we also showed that the Lp-PLA2 concentration was definitely correlated with apoB and apoB/apoA1 after adjustment for hypertension, age, gender, smoking, and diabetes mellitus.

Several limitations upon the current research were proposed as following. Firstly, the single-center samples can be one important limitation to explore the relationships among diverse variable analyses. Secondly, the conclusion tends to be less convinced due to the relatively low coefficients, although the relation between the Lp-PLA2 concentrations and apolipoprotein particles had been statistically significant in the current study. Finally, the recruited population accounted for a relatively inhomogeneous cohort in terms of age and gender, which may have biased the conclusions.

## 5. Conclusion

We revealed that the plasma levels of Lp-PLA2 provide positively a key link with apoB, apoB/apoA-1 among stable CAD, denoting the communication between Lp-PLA2 and apolipoprotein particles in the state of CAD. It is still needed to evaluate the specific mechanism by further studies.

## Figures and Tables

**Table 1:** Baseline features of the study population.

	Non-CAD (*n* = 278)	Stable CAD (*n* = 291)	*P*
Sex, M/F	131/147	148/143	0.373
Age, y	63.3 ± 13.0	69.0 ± 11.0	<0.001
Hypertension, *n* (%)	191 (68.7)	232 (79.7)	0.003
Diabetes, *n* (%)	49 (17.6)	79 (27.1)	0.014
Smoking, *n* (%)	103 (37.1)	158 (54.3)	0.096
BMI, kg/m^2^	23.9 ± 1.7	25.9 ± 2.2	0.124
Alcohol intake, *n* (%)	133(47.8)	149(51.2)	0.324
Physical inactivity, *n* (%)	121(43.5)	134(46.0)	0.368
DEI/day, (kcal/d)	1986.3 ± 710.2	2032 ± 724.6	0.238
PSS score	19.7 ± 8.2	21.2 ± 8.6	0.274
TC, mmol/L	4.6 ± 1.3	4.3 ± 1.4	0.022
TG, mmol/L	1.9 ± 1.3	1.5 ± 0.7	0.143
LDL, mmol/L	2.8 ± 2.1	2.6 ± 1.6	0.163
HDL, mmol/L	1.2 ± 0.4	1.3 ± 0.3	0.626
ApoA1, g/L	1.5 ± 0.9	1.1 ± 0.2	0.312
ApoB, g/L	0.8 ± 0.2	0.9 ± 0.3	0.370
ApoB/ApoA1	0.76 ± 0.20	0.73 ± 0.22	0.050
TB, umol/L	14.5 ± 10.3	14.0 ± 8.2	0.521
DB, umol/L	3.8 ± 3.5	4.0 ± 3.3	0.614
ALT, U/L	31.2 ± 7.3	29.2 ± 8.1	0.767
AST, U/L	23.0 ± 7.2	24.3 ± 3.0	0.663
Albumin, g/L	39.4 ± 5.4	38.6 ± 5.1	0.061
Scr, umol/L	94.2 ± 48.8	94.3 ± 34.4	0.966
BUN, mmol/L	6.0 ± 3.0	5.9 ± 2.7	0.522
WBC, ^∗^10^9/L	6.8 ± 3.3	6.7 ± 2.1	0.633
RBC, ^∗^10^12/L	4.3 ± 0.5	4.5 ± 0.5	0.286
LP-PLA2, ng/mL	113.2 ± 65.6	136.0 ± 60.5	<0.001

WBC: white blood cell; BMI: body mass index; DEI: dietary energy intake; PSS: perceived stress scale; RBC: red blood cell; TC: total cholesterol; TG: triglyceride; LDL: low-density lipoprotein; HDL: high-density lipoprotein; Scr: serum creatinine; BUN: blood urea nitrogen; TB: total bilirubin; DB: direct bilirubin.

**Table 2:** Multiple linear regression showed the independent associations of the Lp-PLA2 concentrations with the apolipoprotein in stable CAD group.

	Coefficients	*P* value	Adjusted coefficients	*P* value
ApoA1	-0.099	0.121	-0.128	0.245
ApoB	0.390	<0.001	0.364	<0.001
ApoB/ApoA1	0.450	<0.001	0.390	<0.001

## Data Availability

The data used to support the findings of this study are available from the corresponding author upon request.
